# Co-Packaged PARP inhibitor and photosensitizer for targeted photo-chemotherapy of 3D ovarian cancer spheroids

**DOI:** 10.1186/s13578-024-01197-6

**Published:** 2024-02-06

**Authors:** Aaron Sorrin, Anika Dasgupta, Kathryn McNaughton, Carla Arnau Del Valle, Keri Zhou, Cindy Liu, Dana M. Roque, Huang Chiao Huang

**Affiliations:** 1 Fischell Department of Bioengineering, University of Maryland, College Park, MD 20742 USA; 2 Department of Obstetrics, Gynecology, and Reproductive Sciences, University of Maryland School of Medicine, Baltimore, MD 21201 USA; 3 Greenebaum Comprehensive Cancer Center, University of Maryland, Baltimore, MD 21201 USA

**Keywords:** Photodynamic therapy, Photoimmunotherapy, PARP inhibitor, 3D spheroid, Cancer organoid, Polymeric nanoparticles, Ovarian cancer

## Abstract

**Background:**

Within the last decade, poly(ADP-ribose) polymerase inhibitors (PARPi) have emerged in the clinic as an effective treatment for numerous malignancies. Preclinical data have demonstrated powerful combination effects of PARPi paired with photodynamic therapy (PDT), which involves light-activation of specialized dyes (photosensitizers) to stimulate cancer cell death through reactive oxygen species generation.

**Results:**

In this report, the most potent clinical PARP inhibitor, talazoparib, is loaded into the core of a polymeric nanoparticle (NP-Tal), which is interfaced with antibody-photosensitizer conjugates (photoimmunoconjugates, PICs) to form PIC-NP-Tal. In parallel, a new 3D fluorescent coculture model is developed using the parental OVCAR-8-DsRed2 and the chemo-resistant subline, NCI/ADR-RES-EGFP. This model enables quantification of trends in the evolutionary dynamics of acquired chemoresistance in response to various treatment regimes. Results reveal that at a low dosage (0.01 μM), NP-Tal kills the parental cells while sparing the chemo-resistant subline, thereby driving chemoresistance. Next, PIC-NP-Tal and relevant controls are evaluated in the 3D coculture model at multiple irradiation doses to characterize effects on total spheroid ablation and relative changes in parental and subline cell population dynamics. Total spheroid ablation data shows potent combination effects when PIC and NP-Tal are co-administered, but decreased efficacy with the conjugated formulation (PIC-NP-Tal). Analysis of cell population dynamics reveals that PIC, BPD + NP-Tal, PIC + NP-Tal, and PIC-NP-Tal demonstrate selection pressures towards chemoresistance.

**Conclusions:**

This study provides key insights into manufacturing parameters for PARPi-loaded nanoparticles, as well as the potential role of PDT-based combination therapies in the context of acquired drug resistance.

**Supplementary Information:**

The online version contains supplementary material available at 10.1186/s13578-024-01197-6.

## Background

The poly(ADP-ribose) (PAR) post-translational modification is a major biological regulator with broad roles in cell survival, gene expression, and energy metabolism [1]. The transfer of PAR chains to target proteins is accomplished by PAR polymerases (PARPs), which use NAD^+^ as a substrate and generate nicotinamide as a byproduct. PARP-1 binds to DNA single-strand breaks (SSBs), initiating PARylation of acceptor proteins including PARP-1, histones, and other DNA repair proteins [2]. These appended PAR chains recruit additional DNA repair molecules for SSB rectification, such as X-ray repair cross-complementing protein 1 (XRCC1) [2, 3]. In recent years, there has been increasing clinical interest in PARP inhibitors (PARPi) for oncologic applications, particularly in patients with *BRCA* mutations where synthetic lethality can be achieved [4]. Mechanistically, PARPi function through (1) directly competing with NAD^+^ at the PARP catalytic site and (2) trapping PARP at the SSB site, forming toxic PARP-DNA complexes [5].

PARPi first entered the clinical sphere in 2014 with the United States Food and Drug Administration (FDA) and European Medicines Agency (EMA) approvals of olaparib for the treatment of advanced ovarian cancer [4]. Since then, three additional PARPi (rucaparib, niraparib, and talazoparib) have been FDA-approved for clinical use. These agents have been used for the treatment of numerous malignancies including ovarian, breast, pancreatic, prostate, fallopian, and primary peritoneal cancers [4]. Talazoparib, the most recently FDA-approved PARPi, exhibits the greatest potency compared to olaparib, rucaparib, and niraparib, with lower half maximal inhibitory concentration (IC_50_) values for PARPs-1, -2, -3, -4, and the strongest PARP trapping capabilities [6, 7]. While talazoparib is the most potent PARPi, it is also the most toxic to normal cells and the most poorly tolerated. As a result, maximum tolerated dose of talazoparib is at least 300-fold lower than that of other clinically used PARPi [8]. Combinational therapeutic strategies are a cornerstone in cancer therapeutics that may be leveraged to enable dose reductions of the individual therapies while maximizing anti-cancer effects [9]. In this study, a novel nanoplatform is engineered for ovarian cancer-targeted codelivery of talazoparib with photodynamic therapy (PDT).

PDT involves the light-activation of photosensitive dyes (photosensitizers) resulting in the generation of reactive molecular species which can induce direct cytotoxicity and modulate biological processes [10]. Prior work has established harmonization between PDT and PARPi as an anti-cancer combination regimen for applications in ovarian, gastric, pancreatic, and skin cancers [11–14]. Tanaka et al. found that talaporfin-mediated PDT enhanced PARP-trapping capabilities of olaparib; and their combination significantly suppressed gastric tumor growth in a xenograft murine model [12]. Lei et al. codelivered chlorin e6 and olaparib to pancreatic cancer cells and demonstrated that their combination enhanced cytotoxicity, reactive oxygen species generation, and DNA damage [13]. We have previously demonstrated that olaparib in combination with PDT using benzoporphyrin derivative (BPD) effectively reduced survival and clonogenicity of a coculture system of chemo-sensitive and chemo-resistant ovarian cancer cells [11]. In the same study, we further demonstrated that a lipidated photosensitizer formulation reduced selective survival advantage of the chemo-resistant cells, effectively redirecting cancer evolution dynamics [11]. This exemplifies the potential of nanoengineered combination therapies for overcoming critical barriers to clinical translation such as multidrug resistance. This is particularly relevant for PARP inhibitors, which have been shown in preclinical studies to induce acquired drug resistance through overexpression of multidrug resistance protein 1 (MDR1, P-gp, ABCB1). Rottenberg et al. compared olaparib-sensitive and olaparib-resistant tumors by quantifying the *abcb1a/b* genes that encode for murine P-gp and found up to 85-fold increase in over 70% of resistant tumors compared to those sensitive [15]. They further demonstrated that olaparib resistance could be reversed by the addition of a P-gp inhibitor, tariquidar. In another study, Oplustilova et al. showed, using a proliferation assay, that the P-gp inhibitor verapamil sensitized HCT116 colon cancer cells to PARP inhibitor KU 58948 [16].

In this study, nanoengineering approaches are leveraged for encapsulation of talazoparib in polymeric nanoparticles (NP-Tal). NP-Tal is surface-decorated with antibody-photosensitizer conjugates (photoimmunoconjugates, PIC) using click chemistry for dual functionalization with cancer-targeting capabilities and PDT (PIC-NP-Tal). Optimal synthesis parameters are established to determine the masses of polymer and talazoparib added to the synthesis, as well as PIC-to-nanoparticle ratio. In parallel, a three-dimensional coculture model of fluorescently labelled ovarian cancer cells is developed to examine evolution of multi-drug resistance. The model is comprised of the parental OVCAR8-DsRed2 cells grown with their chemo-resistant P-gp overexpressing subline, NCI/ADR-RES-EGFP. This model enables fluorescence-based longitudinal viability tracking of each cell line in response to treatment, potentiating precise dose optimization. Results demonstrate that low dose NP-Tal (0.01 μM) trends towards selection of the drug-resistant populations by killing the parental OVCAR8-DsRed2 but sparing the NCI/ADR-RES-EGFP subline. In contrast, higher doses of NP-Tal (> 0.01 μM) kill both cell lines to similar degrees. Next, the combination of PIC and NP-Tal is compared to the conjugated PIC-NP-Tal to evaluate the role of conjugation on therapeutic effect. Results demonstrate potent combination effects of PIC and NP-Tal when mixed, but less potent effects when conjugated together. Additionally, treatment with PIC, BPD + NP-Tal, PIC + NP-Tal, and PIC-NP-Tal demonstrated selection pressures for the chemo-resistant subline, whereas NP-Tal alone and BPD alone kill both cell lines to equivalent degrees across all light doses tested. Results from this work provide fundamental implications for the combination of photoimmunotherapy (PIT) and PARP inhibition in the context of drug-resistant ovarian cancer.

## Methods

### Synthesis of photoimmunoconjugates

PIC synthesis was performed by adapting our previous protocols [17, 18]. First, 10 kDa methoxy PEG succinimidyl carboxymethyl ester (mPEG-NHS; JenKem Technology) was added dropwise to Cetuximab at a 3:1 molar ratio and reacted overnight under continuous stirring at room temperature. Next, BPD *N*-hydroxysuccinimidyl ester (BPD-NHS) and azide-PEG4-*N*-hydroxysuccinimidyl ester (azide-PEG-NHS; Thermo Scientific) were added to the reaction to a final ratio of 9 and 2.5 mol per 1 mol Cetuximab, respectively. After another 20 h of stirring at room temperature, the mixture was purified using a 30 kDa Zeba spin desalting column (7 kDa MWCO; Thermo Scientific) and concentrated using an Amicon centrifugal filter unit (30 kDa MWCO, Millipore Sigma). Final Cetuximab concentration was determined by Pierce™ BCA Protein Assay Kit (ThermoFisher Scientific), and final BPD concentration was determined by UV–vis spectroscopy.

### Synthesis of PIC-functionalized polymeric nanoparticles

For talazoparib-loaded nanoparticles (NP-Tal), synthesis parameters were initially varied for protocol optimization (Table 1). PLGA-PEG-COOH and PLGA-PEG-DBCO were obtained from PolySciTech, and talazoparib (Tal) was obtained from MedChemExpress. Polymer was first co-dissolved with talazoparib in 1 mL of acetone, then added to 10 mL of ultrapure water (Invitrogen) containing 0.1% Pluronic F-68 (Gibco). The solution was sonicated with a 120 Watt, 20 kHz probe sonicator at 20% amplitude for 3 min and acetone was evaporated at room temperature for 4–6 h under continuous stirring at 400 rpm. The obtained NP-Tal were filtered through 0.22 μm syringe filter units (Millipore) and concentrated in an Amicon centrifugal filter unit (30 kDa MWCO, Millipore Sigma). Next, PIC was conjugated to the DBCO-containing nanoparticles through copper-free click chemistry. For conjugation, PIC and nanoparticles were mixed overnight at volume ratios of 0.5:1, 1:1, 2:1, and 3:1, then purified via Sepharose CL-4B size exclusion chromatography.

**Table 1:** Varying parameters in nanoparticle formulation

Parameter	Values
PLGA-PEG-COOH (mg)	10.7, 21.4, 42.8, 85.6
Talazoparib (mg)	0, 0.107, 0.535, 1.07, 2.675
PLGA-PEG-DBCO/total polymer (%)	0, 25, 50, 100

### Photophysical and photochemical nanoparticle characterization

Talazoparib concentration was determined using a fluorescence-based standard curve (ex/em; 312/416 nm, Synergy neo2, Biotek). BPD concentration was calculated similarly (ex/em; 435/700 nm). Loading capacity (%) was calculated as the mass of polymer divided by the mass of loaded talazoparib. The talazoparib encapsulation efficiency (%) was calculated as the ratio of nanoparticle-loaded talazoparib to the initial talazoparib added to the nanoparticle synthesis reaction. Talazoparib retention (%) was calculated as the ratio of talazoparib after and before PIC conjugation. PIC conjugation efficiency (%) was determined by calculating the ratio of BPD loaded onto the nanoparticle to the initial BPD added to the conjugation reaction. PIC per nanoparticle was calculated by first determining molecules of PIC using Pierce™ BCA Protein Assay Kit (ThermoFisher Scientific), then dividing by the number of nanoparticles as determined by NanoSight LM10 (Malvern Instruments). Talazoparib per nanoparticle was calculated as molecules of talazoparib divided by number of nanoparticles. Nanoparticle size, polydispersity index, and zeta potential were determined using the Nanobrook Omni (Brookhaven Instruments). To quantify photoactivity, compounds were dissolved in PBS or DMSO, then fluorescence emission was collected upon light-activation at 435 nm. Maximum fluorescence emission in PBS was divided by maximum fluorescence emission in DMSO for photoactivity values. Singlet oxygen generation was determined using the Singlet Oxygen Sensor Green (SOSG) probe (Invitrogen). Selectivity and uptake studies of PIC versus PIC-NP-Tal were performed with OVCAR8 (EGFR +) and J774 (EGFR-) cells. First, 300,000 cells were plated and incubated overnight. Next, dishes were treated with 1 μM PIC or PIC-NP-Tal for 30 min. Cells were lysed using radioimmunoprecipitation assay buffer, then BPD fluorescence was measured at ex/em 435/700 nm.

### 3D ovarian cancer coculture system development and treatment regimen

High grade serous ovarian cancer cell lines OVCAR8-DsRed2 and NCI/ADR-RES-EGFP were obtained courtesy of Dr. Michael M. Gottesman (National Cancer Institute, National Institutes of Health). Both cell lines were cultured in RPMI-1640 medium (Corning) supplemented with 10% fetal bovine serum (Gibco), 100 U/mL penicillin and 100 μg/mL streptomycin (Corning). Every four passages, media was supplemented with G418 (Invitrogen) at 500 μg/mL (OVCAR8-DsRed2) or 200 μg/mL (NCI-ADR-RES-EGFP). The growth dynamics of these cell lines on 2D substrates was described previously by our group [11]. In the present study, 3D spheroidal cocultures were generated by plating equal numbers of OVCAR8-DsRed2 and NCI/ADR-RES-EGFP to a final cell number of 1000, 2000, or 5000 cells per well in ultra-low-attachment, round bottom 96 well plates (PerkinElmer). The Lionheart FX Automated Microscope (Biotek) was used for imaging 4 h after plating, 24 h after plating, and then every two days up to day 12. For treatment evaluation, 2,000 cells (1000 of each cell line) were treated on day 4 for 24 h prior to light activation (690 nm, Modulight, Inc.) on day 5. Longitudinal imaging was conducted as described above, and final cell viability analysis was conducted on day 12 using the CellTiter-Glo^®^ Cell Viability Assay (Promega). Total killing controls were achieved by treating spheroids on day 12 with 5% bleach for four hours prior to viability analysis.

### Statistical analysis

GraphPad Prism version 9.0.2 was used for statistical analysis. All data shown were collected at least in triplicate and plotted as mean ± standard error of the mean. Details regarding statistical testing are elaborated in figure captions, and statistical significance was determined as P < 0.05.

## Results

### Development of talazoparib (Tal)-loaded polymeric nanoparticles

**Fig. 1:**
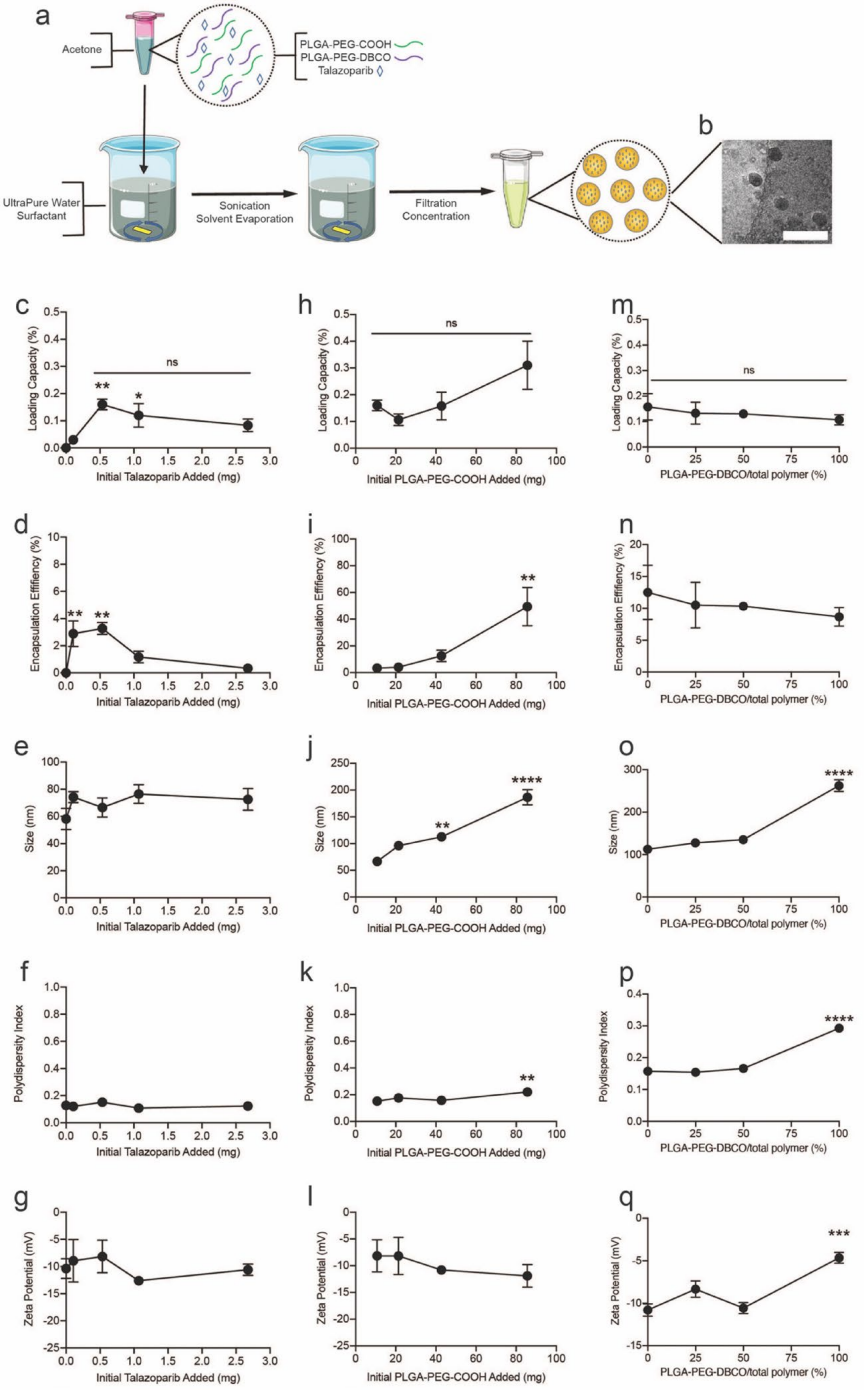
Schematic and characterization of talazoparib-loaded nanoparticles. NP-Tal is prepared by co-dissolution of polymers and talazoparib in acetone, which is added into water containing 0.1% Pluronic F-68 surfactant. The mixture is sonicated, and solvent is evaporated under constant spinning for 4–6 h, then nanoparticles are concentrated using 30 kDa MWCO centrifugal filter (**a**). Representative TEM images are shown (**b**). Scale bar: 250 nm. During optimization procedures, initial talazoparib (mg) was varied (**c**-**g**) and initial PLGA-PEG-COOH (mg) was varied (**h**–**l**). (**m**-**q**) Next PLGA-PEG-DBCO was mixed in with PLGA-PEG-COOH at varying amounts from 0–100% where total polymer mass remained fixed at 42.8 mg. The characterized parameters include loading capacity (%), encapsulation efficiency (%), size (nm), polydispersity index, and zeta potential. * *p* ≤ 0.05; ** *p* ≤ 0.01; *** *p* ≤ 0.001*;* **** *p* ≤ 0.0001

NP-Tal were prepared by nanoprecipitation methods, where acetone and ultrapure water were used for the organic and aqueous phase, respectively (Fig. 1a). A representative TEM image of the NP-Tal is shown in Fig. 1b. The development of nanoparticle synthesis optimization began with varying the initial amount of talazoparib added to the reaction (Fig. 1c–g; Additional file 1: Fig S1). For these studies, the amount of polymer (PLGA-PEG-COOH) added to the reaction was fixed at 10.7 mg. Tested masses of talazoparib included 0, 0.107, 0.535, 1.070, and 2.675 mg. The loading capacity was first measured (Fig. 1c). Loading capacity initially increases, then plateaus, where a maximum is reached at 0.535 mg. This is further shown by encapsulation efficiency data (Fig. 1d), calculated as the percent of initially added talazoparib that is successfully encapsulated in the nanoparticles. Encapsulation efficiency is consistently at ~ 3% when 0.107 and 0.535 mg are added (*p* < 0.01), then decreases at higher values. NP-Tal are ~ 15 nm larger than empty nanoparticles (Fig. 1e), and sizes remain stable across formulations for up to 24 weeks (Additional file 1: Fig S1a). Polydispersity index (PdI) is a measure of the heterogeneity of particle size, and it was recorded for all formulations using a dynamic light scattering (DLS) instrument. PdI of all formulations was initially below 0.16 (Fig. 1f) and remained stable at or below 0.21 for up to 24 weeks (Additional file 1: Fig S1b). Zeta potentials (Fig. 1g) remain relatively consistent across batches. For subsequent reactions, 0.535 mg was set as the talazoparib mass due to maximum loading being reached.

Next, the mass of PLGA-PEG-COOH added in the nanoparticle synthesis reaction was varied (Fig. 1h–l). The initial mass of talazoparib was fixed at 0.535 mg, and polymer was added at 10.7, 21.4, 42.8 or 85.6 mg. No significant changes in loading capacity were calculated (Fig. 1h), encapsulation efficiency increased with increasing polymer mass (Fig. 1i), and size increased significantly for nanoparticles made with 42.8 and 85.6 mg polymer (Fig. 1j). Up to 24 weeks, size of all groups remained stable (Additional file 1: Figure S2a). The PdI remained relatively consistent (~ 0.16) at 10.7, 21.4, 42.8 mg polymer but increased significantly at 85.6 mg polymer to 0.22 (*p* < 0.01) (Fig. 1k). PdI for all groups remained consistent for up to 24 weeks (Additional file 1: Figure S2b). Zeta potential remained relatively consistent across formulations (Fig. 1l). Based on these results, 42.8 mg polymer was selected as the optimal mass of polymer due to the significant increase in PdI at 85.6 mg.

In order to later ‘click’ azide-functionalized photoimmunoconjugates (PIC) onto the nanoparticle, PLGA-PEG-DBCO was incorporated into the formulation. PLGA-PEG-DBCO was mixed with PLGA-PEG-COOH to a final total polymer mass of 42.8 mg, and the relative mass of PLGA-PEG-DBCO was varied from 0, 25, 50, and 100% (Fig. 1m–q). The talazoparib loading capacity and encapsulation efficiency remained relatively consistent with increasing mass of PLGA-PEG-DBCO (Fig. 1m–n). In contrast, size, PdI, and zeta potential increased significantly when nanoparticles were prepared with 100% PLGA-PEG-DBCO (Fig. 1o–q). Size and PdI remained stable across 24 weeks for groups where the mass percent of PLGA-PEG-DBCO was below 100% (Additional file 1: Fig S3a, b). However, for 100% PLGA-PEG-DBCO nanoparticles, after 24 weeks, size decreased from ~ 300 nm to below 200 nm, and PdI decreased from ~ 0.3 to 0.22. Due to the instability and high PdI of 100% PLGA-PEG-DBCO, the 50% PLGA-PEG-DBCO condition was selected for subsequent experiments.

### Optimization and characterization of PIC-conjugated nanoparticles

**Fig. 2:**
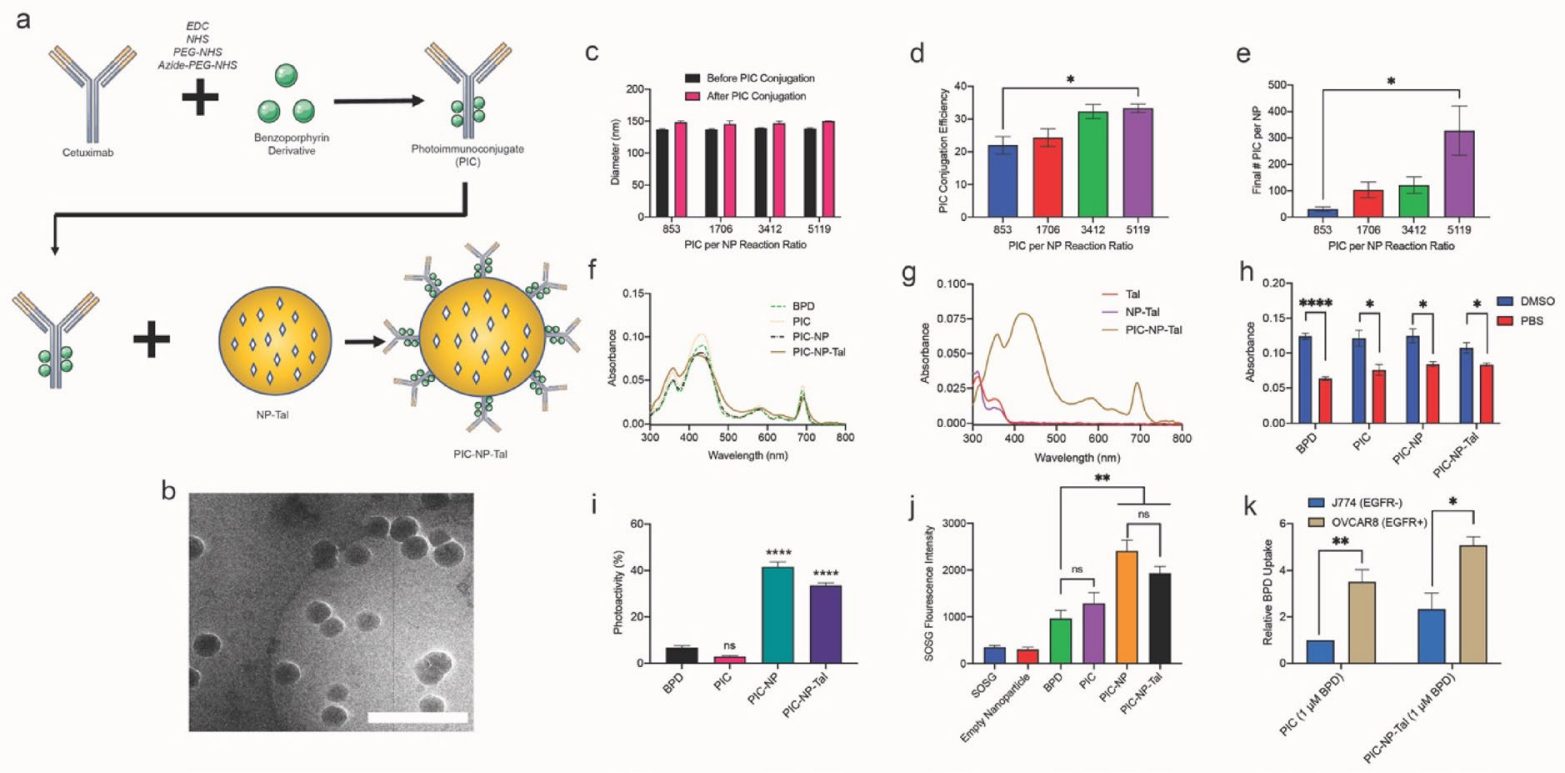
Optimization and characterization of PIC-conjugated nanoparticles. Azide-functionalized photoimmunoconjugates were conjugated to DBCO-containing nanoparticles via copper-free click chemistry (**a**) and visualized by cryoEM (**b**). Scale bar: 500 nm. Volume ratio of PIC:NP was varied and changes in size (**c**), PIC conjugation efficiency (**d**), and number of PICs per nanoparticle (**e**) were characterized. The absorbance spectra are shown for BPD-containing formulations (**f**) and talazoparib-containing formulations (**g**) from 300–800 nm in DMSO. Next, comparison of 690 nm absorbance was performed in DMSO versus PBS (**h**). Photoactivity was evaluated, as described in *Materials & Methods,* for BPD, PIC, PIC-NP, and PIC-NP-Tal (**i**). Singlet oxygen was next quantified using SOSG (Invitrogen) (**j**). Uptake of 1 μM PIC versus 1 μM PIC-NP-Tal in EGFR-negative J774 cells compared to EGFR-positive OVCAR8 cells after 30 min incubation (**k**). Data is normalized to J774 uptake of PIC. * *p* ≤ 0.05; ** *p* ≤ 0.01; *** *p* ≤ 0.001*;* **** *p* ≤ 0.0001

Azide-functionalized PICs composed of Cetuximab and benzoporphyrin derivative (BPD) were ‘clicked’ onto the surface of DBCO-functionalized nanoparticles (Fig. 2a, b). PICs were first prepared using carbodiimide chemistry at a 4:1 final BPD: Cetuximab ratio. Next, 100 μL of nanoparticles were reacted overnight with PIC at varying volumes (50, 100, 200, 300 μL) resulting in volume ratios of 0.5:1, 1:1. 2:1, and 3:1 (PIC: NP), equivalent to PIC per nanoparticle reaction ratios of 853, 1706, 3412, and 5119. PIC-conjugated nanoparticles (PIC-NP) were purified by size exclusion chromatography and characterized for size, PIC conjugation efficiency (based on BPD concentration), and PIC per nanoparticle (Fig. 2c–e). Dynamic light scattering data revealed that PIC conjugation to nanoparticles increased particle diameter by ~ 10 nm (Fig. 2c). PIC conjugation efficiency (amount of photosensitizer conjugated to the nanoparticle relative to the amount added to the synthesis reaction) increased with higher PIC: NP reaction volume ratios, reaching a plateau at the 2:1 volume ratio around ~ 32% (Fig. 2d). Next, the number of PICs per nanoparticle was calculated, revealing a range from ~ 30–330 PIC/NP at varying reaction volumes (Fig. 2e). Due to the plateau in reaction efficiency occurring at a 2:1 PIC: NP volume ratio, this condition was selected for subsequent studies. Stability of PIC-NP and PIC-NP-Tal in size and PdI was confirmed for up to 12 weeks (Additional file 1: Fig S4).

PIC-NP-Tal was next prepared and characterized (Fig. 2f–k, Table 2). Absorbance spectra were recorded (Fig. 2f, g), demonstrating that all BPD-containing agents (BPD, PIC, PIC-NP, PIC-NP-Tal) have the characteristic BPD absorbance peaks at ~ 435 nm and ~ 700 nm. Tal-containing formulations show characteristic absorbance peaks at ~ 312 nm. Next, quenching in aqueous solution was evaluated by comparing absorbance at 690 nm in PBS (quenched) versus DMSO (unquenched) (Fig. 2h). BPD, PIC, PIC-NP, and PIC-NP-Tal all exhibit quenching, shown as significant reductions (~ 20–50%) in absorbance in PBS compared to DMSO. Free BPD and PIC show low photoactivity (< 7%) due to quenching in aqueous solution, whereas the photoactivity of PIC-NP and PIC-NP-Tal is significantly higher, at 42% and 33%, respectively **(**Fig. 2i). In Fig. 2j, singlet oxygen generation based on SOSG fluorescence signal is shown. Compared to BPD, PIC-NP and PIC-NP-Tal show significantly higher fluorescence emission intensity (P < 0.01), representing elevated singlet oxygen yield.

**Table 2:**
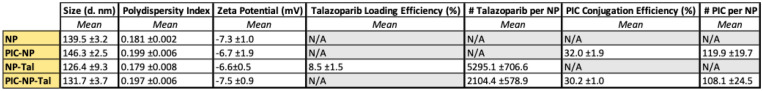
Characterization of nanoparticle physical properties and drug loading

Talazoparib loading efficiency is defined as the moles of talazoparib loaded into the nanoparticle divided by the moles of talazoparib added to the nanoparticle synthesis reaction. # talazoparib per NP is defined as the molecules of talazoparib divided by the number of nanoparticles. PIC conjugation efficiency is defined as the moles of BPD conjugated to the nanoparticle divided by the moles of BPD added to the initial conjugation reaction. # PIC per NP is defined as the molecules of PIC (based on antibody) divided by the number of nanoparticles. Each datapoint is mean ± standard error of the mean, representative of at least four individual nanoparticle batches.

EGFR-dependent uptake was next evaluated by treating EGFR-negative J774 cells and EGFR-positive OVCAR8 cells with PIC or PIC-NP-Tal for 30 min, then collecting cells and quantifying internalized BPD (Fig. 2k). PIC uptake by OVCAR8 cells was significantly (P < 0.01) greater than PIC uptake by J774 cells with a 3.5-fold increase in photosensitizer uptake, demonstrating EGFR-enhanced uptake. For PIC-NP-Tal, uptake by OVCAR8 cells was over double that of J774 (P < 0.05).

Nanoparticle properties are summarized in Table 2. Empty nanoparticles (NP) are ~ 140 nm, whereas talazoparib loaded nanoparticles (NP-Tal) are ~ 126 nm. This difference in size is not statistically significant (P = 0.30), and upon PIC conjugation, both nanoparticles increase in size by 5–7 nm. PdI for empty and talazoparib-loaded nanoparticles is around ~ 0.18, whereas PIC conjugated nanoparticles have PdI just under 0.20. For all formulations, zeta potential is consistently around − 7 mV. Talazoparib loading into nanoparticles was determined as 8.5%, and molecules of talazoparib per nanoparticle were calculated as 5295.1 and 2104.4 for NP-Tal and PIC-NP-Tal, respectively. PIC conjugation efficiency is ~ 30% for PIC-NP and PIC-NP-Tal and the number of PIC per nanoparticle is consistent between both formulations at ~ 115 PIC/NP.

### Development of 3-dimensional ovarian cancer coculture model

**Fig. 3:**
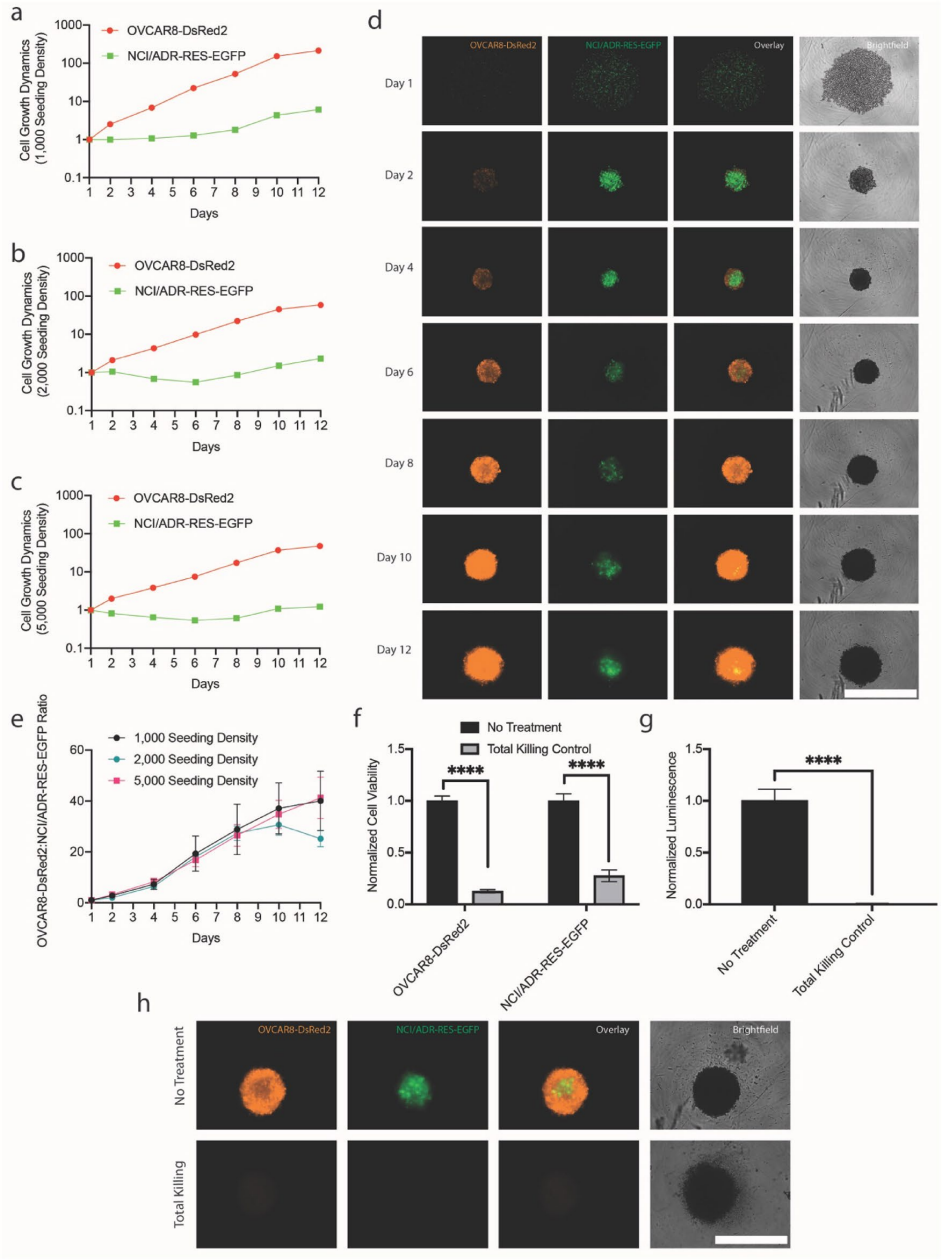
Optimization of 3D coculture model. OVCAR8-DsRed2 and NCI/ADR-RES-EGFP cells were plated at a 1:1 ratio to final seeding cell densities of 1000–5000. Fluorescence signal from cells was recorded up to 12 days and plotted as fold change from day 1 for 1000 (**a**), 2000 (**b**), and 5000 (**c**) cell seeding densities. Representative longitudinal imaging for spheroids with 2000 seeded cells are shown (**d**). Next, fold change in OVCAR8-DsRed2 fluorescence was divided by fold change in NCI/ADR-RES-EGFP fluorescence to calculate the cell growth ratio (**e**). Total killing controls (5% bleach) were included, and viability is plotted as a function of fluorescence (**f**) and CellTiter-Glo® Cell Viability Assay (**g**). Representative images of total killing controls are shown (**h**). Scale bar = 1000 μm. * *p* ≤ 0.05; ** *p* ≤ 0.01; *** *p* ≤ 0.001*;* **** *p* ≤ 0.0001

A 3D coculture model of a parental (OVCAR8-DsRed2) and a chemo-resistant subline (NCI/ADR-RES-EGFP) was developed by seeding 1000, 2000, or 5000 cells at a 1:1 ratio in ultra-low attachment round bottom plates and tracking fluorescence over the course of 12 days (Fig. 3a–c). Representative longitudinal images of spheroids with a 2000 cell seeding density are shown in Fig. 3d. The parental OVCAR8-DsRed2 cells grew drastically faster than NCI/ADR-RES-EGFP at all seeding densities, reaching 48-, 58-, and 215-fold increases in RFU by day 12 in 5000, 2000, and 1000 cell seeding densities, respectively. NCI/ADR-RES-EGFP cells, in contrast reach 1-, 2-, and 6-fold increases in growth by day 12 for 5000, 2000, and 1000 seeding density groups. The growth ratios of OVCAR8-DsRed2:NCI/ADR-RES-EGFP were calculated as the fold change in OVCAR8-DsRed2 RFU relative to day 1 divided by the fold change in NCI/ADR-RES-EGFP RFU relative to day 1 (Fig. 3e). Across all starting seeding densities, this ratio remained relatively consistent over the course of the experiment, with day 12 values at 40, 25, and 41 at 1000, 2000, and 5000 densities, respectively (*P* > 0.4). Fluorescence-based viability tracking was next validated by preparing a total killing control (5% bleach, 4 h) and comparing fluorescence emission intensity values with an ATP-based cell viability assay (CellTiter-Glo® Cell Viability Assay) (Fig. 3f, g). Fluorescence intensity of OVCAR8-DsRed2 and NCI/ADR-RES-EGFP decreased significantly for total killing controls to 13 and 28%, respectively. In contrast, the ATP-based assay showed reductions in viability down to < 1%. The residual fluorescence values for total killing controls are likely resulting from auto-fluorescent contributions. Representative images of total killing controls are shown in Fig. 3h.

### Comparative dosage analysis of NP-Tal in ovarian cancer 3D cocultures

**Fig. 4:**
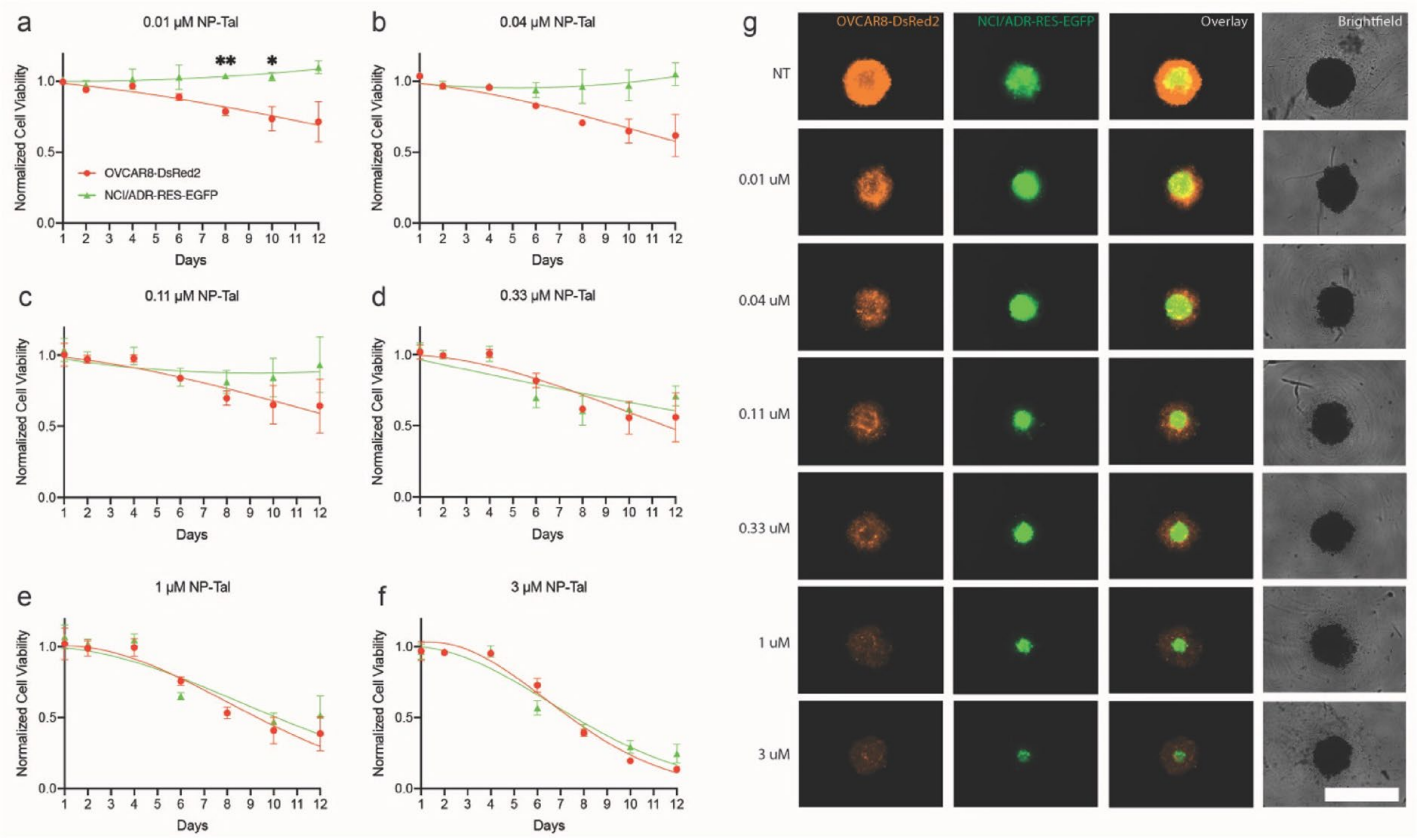
Spheroid toxicity analysis of NP-Tal at varying doses. Spheroids composed of OVCAR8-DsRed2 cells and NCI/ADR-RES-EGFP cells were treated with NP-Tal at varying doses 3 days after seeding. Fluorescence was recorded up to 12 days, and intensity values for each cell line were normalized to the untreated spheroids to calculate cell viability. Longitudinal viability for parental and subline cells are shown from days 1–12 for NP-Tal doses at 0.01 μM (**a**), 0.04 μM (**b**), 0.11 μM (**c**), 0.33 μM (**d**) 1 μM (**e**), and 3 μM (**f**). Representative images of spheroids on day 12 at each treatment dose are shown (**g**). Scale bar = 1000 μm. * *p* ≤ 0.05; ** *p* ≤ 0.01; *** *p* ≤ 0.001*;* **** *p* ≤ 0.0001

Next, treatment effect of NP-Tal in spheroids (2000 cell seeding density) was evaluated using concentrations from 0.01 to 3 μM (Fig. 4). On day 4, when spheroids were fully established, they were treated with varying doses of NP-Tal until day 12. Images were taken longitudinally, and the fluorescence emission intensity of each spheroid was normalized to the untreated spheroid on each respective day to determine viability (Fig. 4a–f). Representative images of spheroids on day 12 are shown (Fig. 4g). Results at the lower NP-Tal doses (0.01–0.11 μM) demonstrate a decrease in viability for the parental cell line whereas the resistant subline was spared (Fig. 4a–c). On the other hand, higher doses (0.33–3 μM) killed both parental and subline cells to a similar degree (Fig. 4d–f). Additional file 1: Fig S5a, b shows fluorescence-based viability analysis of OVACR8-DsRed2 cells and NCI/ADR-RES-EGFP cells, revealing decreases in viability with increasing NP-Tal dosing. On day 12, 3 μM treatment resulted in ~ 14% and ~ 25% viability for OVACR8-DsRed2 cells and NCI/ADR-RES-EGFP cells, respectively. Next, growth curves were calculated based on changes in fluorescence relative to day 1 for each cell line (Additional file 1: Fig S5c, d). Untreated cells show day 12 growth increases at 58-fold and 2-fold for OVACR8-DsRed2 cells and NCI/ADR-RES-EGFP cells, respectively. Increasing NP-Tal dosage caused decreasing fold changes in growth, with day 12 values at 8-fold and 0.6-fold for OVCAR8-DsRed2 cells and NCI/ADR-RES-EGFP cells, respectively, with 3 μM treatment. Dose-dependent effects of NP-Tal are shown in Additional file 1: Fig S6a, b using fluorescence-based and ATP-based viability assays. Fluorescence-based data shows a rightward shift of the NCI/ADR-RES-EGFP cells, representing increased resistance to NP-Tal relative to the parental OVCAR8-DsRed2 cell line.

### PIC-NP-Tal treatment outcomes in 3D spheroid cocultures

**Fig. 5:**
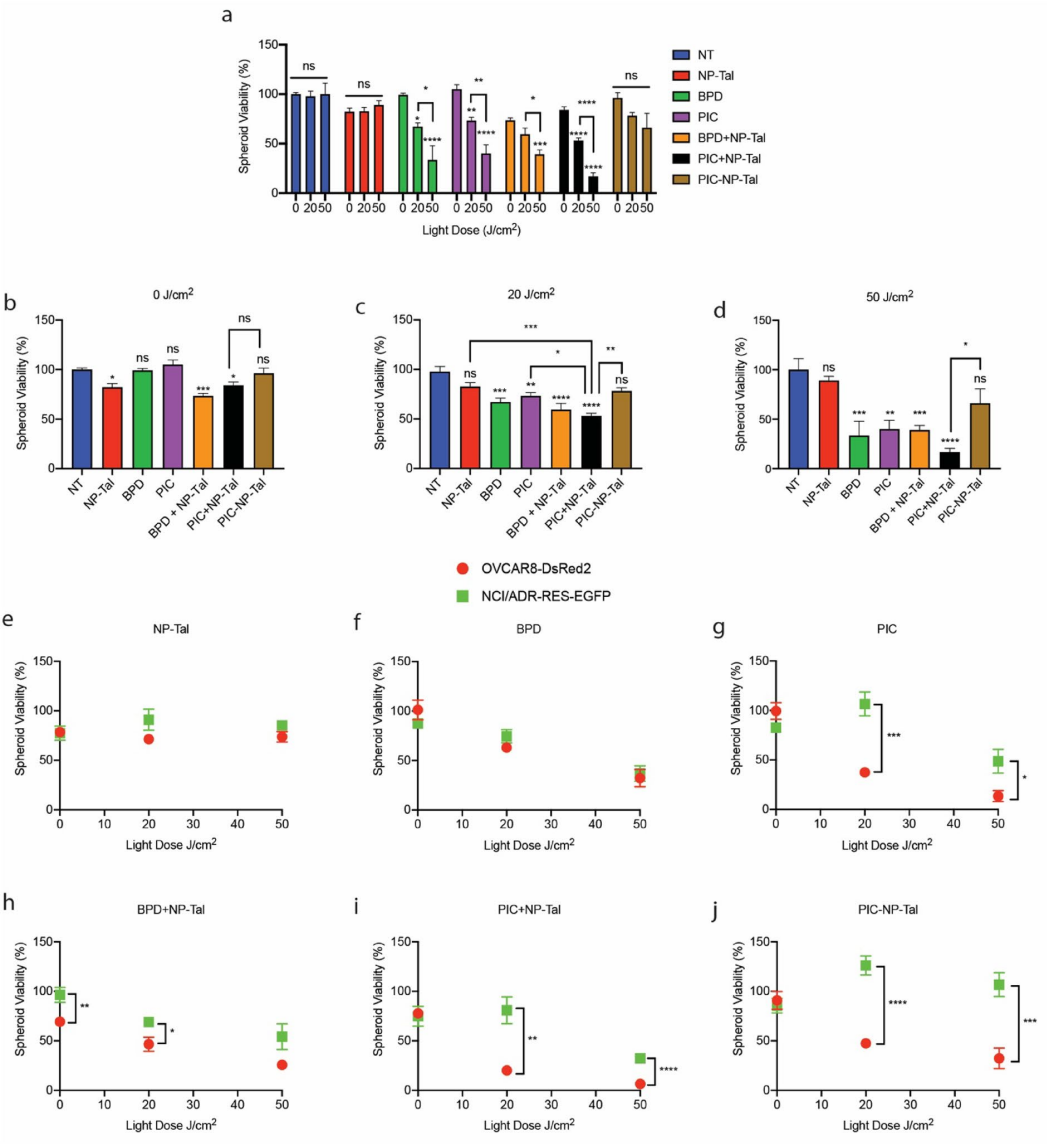
Treatment of 3D cocultures with PIC-NP-Tal. Spheroid cocultures of OVCAR8-DsRed2 and NCI/ADR-RES-EGFP cells were treated with PIC-NP-Tal or relevant controls on day 4. Light-activation was performed at 0 (dark control), 20, or 50 J/cm^2^. Viability analysis using the CellTiter-Glo® Cell Viability Assay was performed, where luminescence values were normalized to the no treatment (NT) 0 J/cm^2^ control (**a**). Normalized luminescence is further analyzed within light doses for 0 (**b**), 20 (**c**), and 50 J/cm^2^ (**d**). Fluorescence-based viability of each cell line, normalized to untreated spheroids, is shown at each light dose for NP-Tal (**e**), BPD (**f**), PIC (**g**), BPD + NP-Tal (**h**), PIC + NP-Tal (**i**), and PIC-NP-Tal (**j**). * *p* ≤ 0.05; ** *p* ≤ 0.01; *** *p* ≤ 0.001; **** *p* ≤ 0.0001

PIC-NP-Tal and monotherapy controls were next tested in the 3D coculture model (Fig. 5). Luminescence-based viability analysis in Fig. 5a shows that there are light-dose dependent effects of BPD, PIC, BPD mixed with NP-Tal (BPD + NP-Tal), and PIC mixed with NP-Tal (PIC + NP-Tal). In contrast, there were no significant light-dose dependent toxicities for the no treatment (NT), NP-Tal, and PIC-NP-Tal groups. Analysis of treatment groups within light doses is shown in Figs. 5b–d. At all light doses, PIC-NP-Tal does not induce significant reductions in viability. In contrast, when PIC and NP-Tal are mixed as an unconjugated pair (PIC + NP-Tal), spheroid viability is reduced to 84% (P < 0.05), 53% (P < 0.0001), and 17% (P < 0.0001) at 0, 20, and 50 J/cm^2^. Notably, at 20 J/cm^2^, PIC + NP-Tal significantly outperforms PIC and NP-Tal alone. BPD alone, PIC alone, and BPD + NP-Tal caused significant reductions in viability at 20 and 50 J/cm^2^, and PIC + NP-Tal significantly outperformed PIC-NP-Tal at 20 and 50 J/cm^2^. Next, parental and subline fluorescence intensities were normalized to untreated spheroids and plotted in Fig. 5e–j. NP-Tal and BPD did not cause significant differences in viability between cell lines across all light doses, demonstrating a lack of selection pressures for either cell line. Interestingly, BPD + NP-Tal does select for chemoresistance, as determined by significantly higher viability of the NCI/ADR-RES-EGFP line compared to the OVCAR8-DsRed2 line at 0 and 20 J/cm^2^. PIC alone, PIC + NP-Tal, and PIC-NP-Tal induce selection pressures towards drug resistance at 20 and 50 J/cm^2^, but not at 0 J/cm^2^.

## Discussion

PARP inhibition has emerged in recent years as a powerhouse chemotherapy for numerous malignancies. In the clinic, PARP inhibitors are used to treat a growing list of indications that currently includes ovarian, breast, pancreatic, prostate, fallopian, and primary peritoneal cancers. Of the currently FDA-approved PARP inhibitors, studies show that talazoparib has the lowest IC_50_ and greatest PARP trapping capabilities. However, talazoparib is also the most toxic, with at least 300-fold lower maximum tolerated dose compared to the other clinically-prescribed PARP inhibitors. Nanoengineering approaches have emerged as a promising strategy to overcome this obstacle and strengthen PARP inhibition as an anti-cancer modality [19–21].

The nanoengineering approach in this study is unique in that it combines talazoparib with photoimmunotherapy to achieve codelivery of PDT and PARP inhibition within a targeted formulation. Work by Spring et al. has demonstrated the capabilities of photoimmunotherapy for ovarian cancer treatment in vivo [22]. They showed that anti-EGFR photoimmunoconjugates composed of Cetuximab and BPD could selectively accumulate in ovarian cancer metastases, enabling precise imaging and treatment. We have previously shown that photoimmunotherapy-functionalized nanoparticles promote enhanced photosensitizer delivery [23] and possess combination-treatment capabilities through co-encapsulation of additional therapeutic entities [17]. Additionally, the clinical relevance of photoimmunotherapy-based anti-cancer approaches has recently been elevated with the clinical use of Cetuximab-IR700 conjugates for the treatment of head and neck cancer in Japan [24].

The present study combines two clinically relevant modalities, photoimmunotherapy and PARP inhibition, in a targeted polymeric nanoparticle for the treatment of ovarian cancer spheroids. First, the nanoformulation was optimized through modulating various synthesis parameters including talazoparib mass, polymer mass, and ratio of two polymers (PLGA-PEG-COOH and PLGA-PEG-DBCO) (Table 1, Fig. 1). The optimized formulation was then functionalized with photoimmunoconjugates via copper-free click chemistry for targeting and photoactivity capabilities (Fig. 2). Consistent with previous work, the final formulation (PIC-NP-Tal) retained the 690 nm Q-band of BPD for light activation and showed superior singlet oxygen generation compared to free BPD [17, 23]. Like PIC, PIC-NP-Tal also demonstrated selectivity for EGFR-expressing cells. Importantly, uptake of PIC-NP-Tal by EGFR-expressing cells was greater than uptake of PIC alone by 45%, demonstrating our previously described “carrier effect” phenomenon in 2-dimensional cultures.

In parallel, we developed a novel fluorescent 3D coculture system of the parental OVCAR8-DsRed2 cells and the drug resistant subline, NCI/ADR-RES-EGFP (Fig. 3). In a previous study, these cell lines were cocultured on 2D substrate, leading to rapid domination of the parental subline [11]. For example, after 7 days, the parental line outnumbered the subline by nearly 5-fold, and by 14 days this difference increased to ~ 20-fold. Similarly, in 3D growth conditions, the parental cell line rapidly outgrows the chemo-resistant subline, and this trend is consistent when cells are plated at varying seeding densities (1000, 2000, 5000 cells per well). Spheroids with lower seeding densities showed greater increases in cellular fluorescence compared to spheroids plated at higher seeding densities, representative of greater spheroid growth (Fig. 3a–c). For example, at the 1000 cell seeding density, the fluorescence emission intensity of OVCAR8-DsRed2 and NCI/ADR-Res-EGFP increased by 215-fold and 6-fold relative to day 1, respectively. In contrast, at the 5000 cell seeding density, OVCAR8-DsRed2 and NCI/ADR-RES-EGFP cell fluorescence changed by 48-fold and 1-fold. However, regardless of seeding density, the parental-to-subline growth ratio remains remarkably consistent throughout the study (Fig. 3e). This method of tracking growth dynamics measures fluorescence intensity at the spheroids’ surface and is limited by this shallow depth of imaging. To further improve this method for future applications, confocal microscopy using z-stack images could be used to gain more information on the whole spheroid.

We next established dose–response studies of NP-Tal in the spheroid coculture model to evaluate the role of talazoparib dose in spheroid evolution (Fig. 4). On the lower dose range, the NCI/ADR-RES-EGFP cell line is spared throughout the study, while the parental OVCAR8-DsRed2 line succumbs to the treatment. In contrast, higher doses kill both cell lines to equivalent degrees. This demonstrates a trend towards acquired chemoresistance where sublethal treatment is applied, a phenomenon consistently observed in prior studies [25]. Next, the PIC-NP-Tal nanocomplex and controls are tested in the 3D coculture model (Fig. 5). At clinically relevant light doses of 20 and 50 J/cm^2^, free BPD alone, PIC alone, and the combination of free BPD with NP-Tal can effectively reduce cancer cell viability. This anti-cancer efficacy can be further enhanced when using the combination of PIC and NP-Tal at 50 J/cm^2^. This superior performance may result from the use of PIC that target delivers BPD to EGFR-positive cancer cells, as well as the synergistic interaction between PDT and PARP inhibitors [11], leading to more effective cancer cell killing. Interestingly, the nanocomplex (PIC-NP-Tal) did not significantly outperform the mixture of PIC with NP-Tal in spheroid killing at 20 and 50 J/cm^2^. Further optimization of PIC-NP-Tal nanocomplex (e.g., PEG and PIC density) and the application of fluorescence-guided strategy [18] to improve the delivery and anti-tumor efficacy of PIC-NP-Tal are warranted. Analyses of the 3D coculture model viability data (Fig. 5e–j, Additional file 1: Fig S8) also revealed that NP-Tal, BPD, and BPD + Nal-Tal at 20 and 50 J/cm^2^ can effectively reduce the viability of both chemo-resistant and chemo-sensitive cancer cell populations. In contrast, treatments with PIC, PIC + NP-Tal, or PIC-NP-Tal at 20 and 50 J/cm^2^ reduced the viability of chemo-sensitive cancer cells, but they did not effectively control the chemo-resistant cells. These results are consistent with our previous findings [26–28], showing that PDT using light-activated BPD mitigates chemo-selection pressure. Further mechanistic studies of the role of PIC-based photoimmunotherapy in modulating chemo-selection pressure in vitro and in vivo are needed.

## Conclusions

Photoimmunotherapy and PARP inhibition are clinically relevant cancer treatment modalities with synergistic potential. In this study, these modalities are combined to achieve a novel nanocomplex for codelivery of Cetuximab-BPD PICs and talazoparib. First, formulation parameters were optimized to establish a polymeric nanoparticle loaded with talazoparib with capabilities for click chemistry to attach PIC. Next, the PIC-to-nanoparticle reaction ratio was optimized, and the formulation was thoroughly characterized for photochemical and biological properties. In parallel, a 3D model of ovarian cancer with fluorescently labeled chemo-sensitive (OVCAR8-DsRed2) and chemo-resistant (NCI/ADR-RES-EGFP) subpopulations was developed and tracked for up to 12 days. Treatment of spheroids with varying doses of NP-Tal revealed that lower doses induce selection pressures in favor of the chemo-resistant subline, whereas higher doses are similarly cytotoxic to both cell lines. Evaluation of PIC-NP-Tal in the 3D spheroid model revealed inferior therapeutic effects compared to co-treatment of PIC and NP-Tal. Additionally, PIC, BPD + NP-Tal, PIC + NP-Tal, and PIC-NP-Tal all drove chemoresistance, whereas NP-Tal and BPD as monotherapies did not. Overall, these data provide new insights into combinational therapies in the context of 3D spheroids, indicating that conjugation of multiple therapeutic entities may not always outperform the unconjugated combination. Results from this study also indicate that while combinational therapies may enhance total cell killing compared to monotherapies, they may also drive chemoresistance, reinforcing the fundamental importance of preclinical models of multidrug resistance.

### Supplementary Information

**Additional file 1: Figure S1.** Stability of nanoparticles with varying initial talazoparib amounts. Polymeric nanoparticles were prepared with 10.7 mg PLGA-PEG-COOH and 0, 0.107, 0.535, or 1.07 mg of talazoparib. Particle size (a) and PdI (b) were tracked longitudinally for up to 24 weeks, with particles stored in ultrapure water at 4°C and protected from light. **Figure S2.** Stability of nanoparticles with varying initial PLGA-PEG-COOH amounts. Polymeric nanoparticles were prepared with 0.535 mg of talazoparib and varied amounts of PLGA-PEG-COOH from 10.7 to 85.6 mg. Particle size (a) and PdI (b) were tracked longitudinally for up to 24 weeks, with particles stored in ultrapure water at 4°C, protected from light. **Figure S3.** Stability of nanoparticles with varying PLGA-PEG-DBCO/total polymer percentages. Polymeric nanoparticles were prepared with 0.535 mg of talazoparib and 42.8 total mg polymer. The polymer component was either PLGA-PEG-COOH, PLGA-PEG-DBCO, or a mixture. Particle size (a) and PdI (b) were tracked longitudinally for up to 24 weeks, with particles stored in ultrapure water at 4°C and protected from light. **Figure S4.** Stability of PIC-conjugated nanoparticles. Polymeric nanoparticles were functionalized with PIC to establish PIC-NP and PIC-NP-Tal formulations. Particles were stored in ultrapure water at 4°C and protected from light. Particle size (a) and PdI (b) were  tracked for 12 weeks. **Figure S5.** Longitudinal spheroid viability and growth tracking. Coculture spheroids were treated with NP-Tal up to 3 μM and imaged on days 1, 2, 4, 6, 8, 10, and 12. Fluorescence values were normalized to untreated cells on each respective day to quantify viability for OVCAR8-DsRed2 cells (a) and NCI/ADR-RES-EGFP cells (b). Growth dynamic of the parental cells (c) and subline (d) are calculated as the fold-change in RFU relative to day 1. **Figure S6.** Day 12 spheroid viability curves. On day 12, coculture spheroids treated with varying doses of NP-Tal were characterized for viability based on fluorescence of the cell lines OVCAR8-DsRed2 and NCI/ADR-RES-EGFP (a) and luminescence of both cell lines in the CellTiter-Glo® Cell Viability Assay (b). **Figure S7.** Stability of nanoparticles in serum. Polymeric nanoparticles, talazoparib-loaded nanoparticles, and polymeric nanoparticles functionalized with PIC (PIC-NP-Tal) were prepared and mixed into calcium- and magnesium-free PBS solution containing 1% fetal bovine serum (FBS). Particles were stored at 37°C and protected from light. Formulations were tracked for 24 hours and particle size (a) and PdI (b) were recorded. **Figure S8.** Treatment of 3D cocultures with PIC-NP-Tal. Fluorescence-based viability of each cell line, normalized to untreated spheroids, is shown at 0 (a), 20 (b) and 50 J/cm (c).

## Data Availability

The datasets used and/or analysed during the current study are available from the corresponding author on reasonable request.

## Abbreviations

BPD: Benzoporphyrin derivative
Cet: Cetuximab
EGFR: Epidermal growth factor receptor
EMA: European Medicines Agency
FDA: United States Food and Drug Administration
NAD^+^: Nicotinamide adenine dinucleotide
NP-Tal: Talazoparib-loaded nanoparticle
PARP: Poly(ADP-ribose) polymerase
PARPi: Poly(ADP-ribose) polymerase inhibitor
PIC: Photoimmunoconjugate
PIC-NP-Tal: Photoimmunoconjugate-functionalized talazoparib-loaded nanoparticle
SSB: Single strand break
XRCC1: X-ray repair cross-complementing protein 1
