# Supplementary material for: Co-Packaged PARP inhibitor and photosensitizer for targeted photo-chemotherapy of 3D ovarian cancer spheroids
Source: Cell Biosci. 2024 Feb 6;14:20. doi: 10.1186/s13578-024-01197-6 (PMC10845736; doi:10.1186/s13578-024-01197-6)
Supplement: Supplementary file 1 — Additional file 1: Figure S1. Stability of nanoparticles with varying initial talazoparib amounts. Polymeric nanoparticles were prepared with 10.7 mg PLGA-PEG-COOH and 0, 0.107, 0.535, or 1.07 mg of talazoparib. Particle size (a) and PdI (b) were tracked longitudinally for up to 24 weeks, with particles stored in ultrapure water at 4°C and protected from light. Figure S2. Stability of nanoparticles with varying initial PLGA-PEG-COOH amounts. Polymeric nanoparticles were prepared with 0.535 mg of talazoparib and varied amounts of PLGA-PEG-COOH from 10.7 to 85.6 mg. Particle size (a) and PdI (b) were tracked longitudinally for up to 24 weeks, with particles stored in ultrapure water at 4°C, protected from light. Figure S3. Stability of nanoparticles with varying PLGA-PEG-DBCO/total polymer percentages. Polymeric nanoparticles were prepared with 0.535 mg of talazoparib and 42.8 total mg polymer. The polymer component was either PLGA-PEG-COOH, PLGA-PEG-DBCO, or a mixture. Particle size (a) and PdI (b) were tracked longitudinally for up to 24 weeks, with particles stored in ultrapure water at 4°C and protected from light. Figure S4. Stability of PIC-conjugated nanoparticles. Polymeric nanoparticles were functionalized with PIC to establish PIC-NP and PIC-NP-Tal formulations. Particles were stored in ultrapure water at 4°C and protected from light. Particle size (a) and PdI (b) were tracked for 12 weeks. Figure S5. Longitudinal spheroid viability and growth tracking. Coculture spheroids were treated with NP-Tal up to 3 μM and imaged on days 1, 2, 4, 6, 8, 10, and 12. Fluorescence values were normalized to untreated cells on each respective day to quantify viability for OVCAR8-DsRed2 cells (a) and NCI/ADR-RES-EGFP cells (b). Growth dynamic of the parental cells (c) and subline (d) are calculated as the fold-change in RFU relative to day 1. Figure S6. Day 12 spheroid viability curves. On day 12, coculture spheroids treated with varying doses of NP-Tal were characterized for [file 13578_2024_1197_MOESM1_ESM.docx]

Additional File Figures


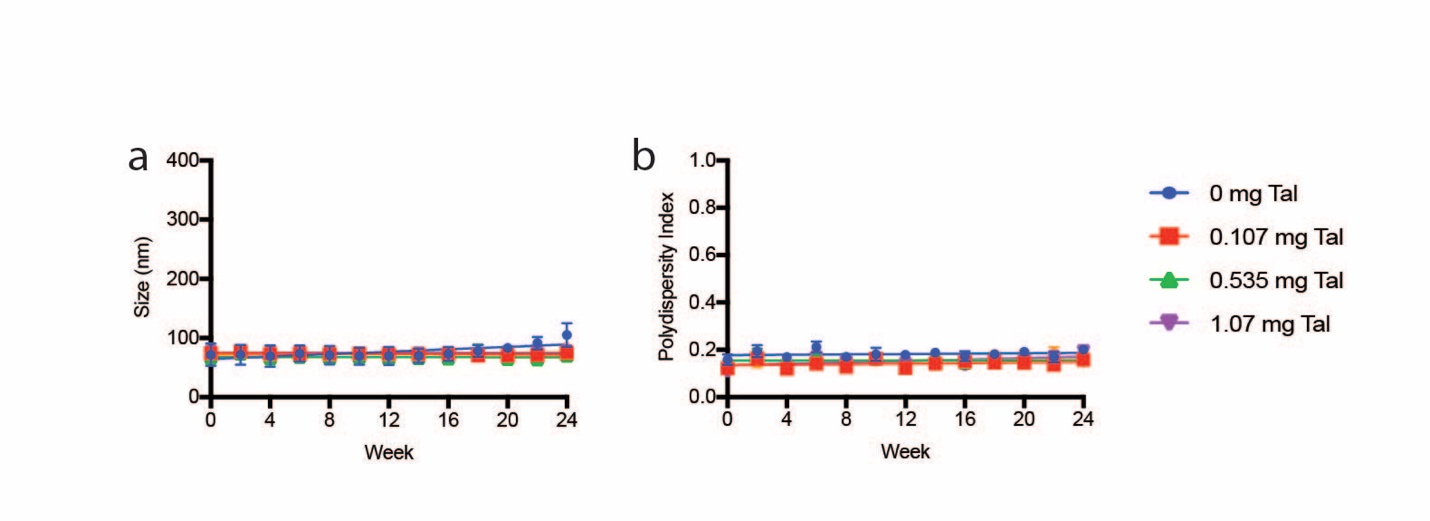


**Figure S1: Stability of nanoparticles with varying initial talazoparib amounts.** Polymeric nanoparticles were prepared with 10.7 mg PLGA-PEG-COOH and 0, 0.107, 0.535, or 1.07 mg of talazoparib. Particle size (**a**) and PdI (**b**) were tracked longitudinally for up to 24 weeks, with particles stored in ultrapure water at 4°C and protected from light.


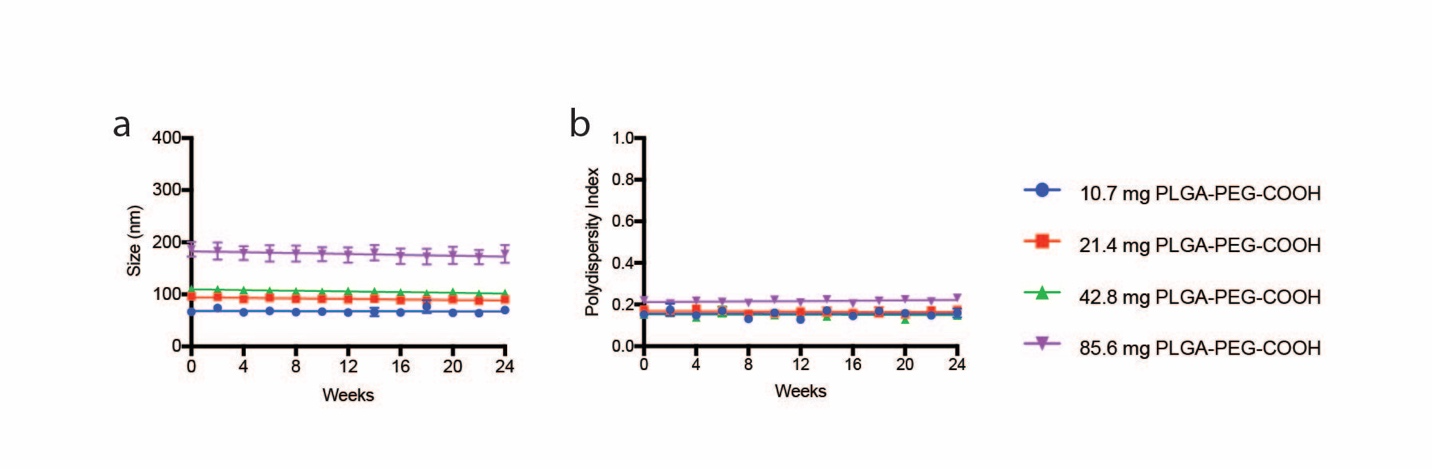


**Figure S2: Stability of nanoparticles with varying initial PLGA-PEG-COOH amounts.** Polymeric nanoparticles were prepared with 0.535 mg of talazoparib and varied amounts of PLGA-PEG-COOH from 10.7 to 85.6 mg. Particle size (**a**) and PdI (**b**) were tracked longitudinally for up to 24 weeks, with particles stored in ultrapure water at 4°C, protected from light.


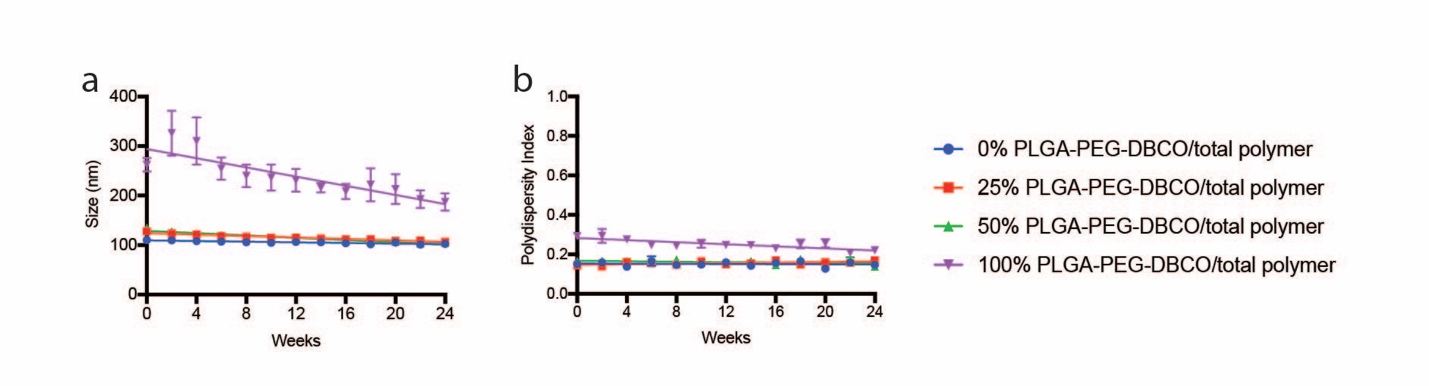


**Figure S3: Stability of nanoparticles with varying PLGA-PEG-DBCO/total polymer percentages.** Polymeric nanoparticles were prepared with 0.535 mg of talazoparib and 42.8 total mg polymer. The polymer component was either PLGA-PEG-COOH, PLGA-PEG-DBCO, or a mixture. Particle size (**a**) and PdI (**b**) were tracked longitudinally for up to 24 weeks, with particles stored in ultrapure water at 4°C and protected from light.

**Figure S4: Stability of PIC-conjugated nanoparticles.** Polymeric nanoparticles were functionalized with PIC to establish PIC-NP and PIC-NP-Tal formulations. Particles were stored in ultrapure water at 4°C and protected from light. Particle size (**a**) and PdI (**b**) were tracked for 12 weeks.


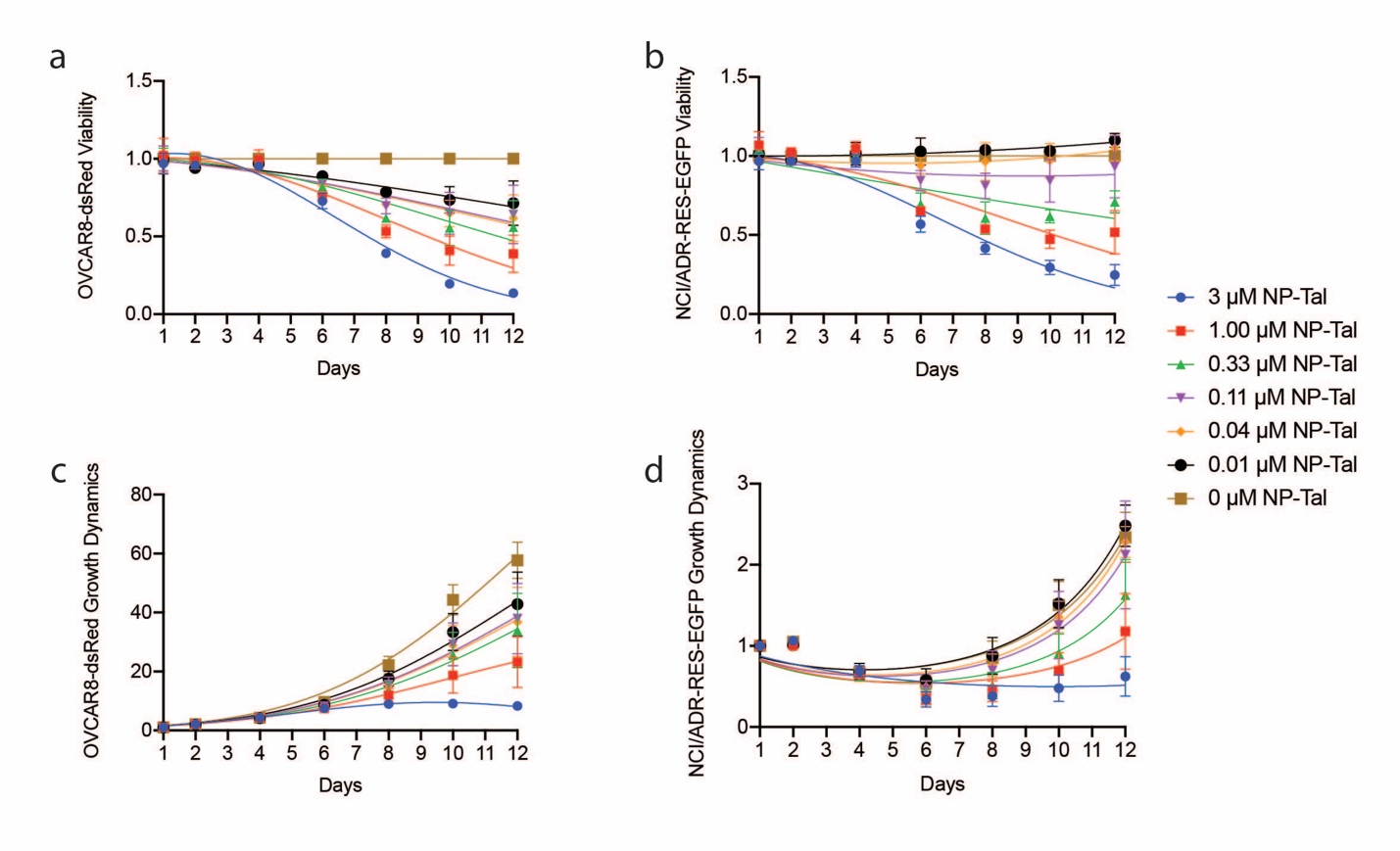


**Figure S5: Longitudinal spheroid viability and growth tracking.** Coculture spheroids were treated with NP-Tal up to 3 μM and imaged on days 1, 2, 4, 6, 8, 10, and 12. Fluorescence values were normalized to untreated cells on each respective day to quantify viability for OVCAR8-DsRed2 cells (**a**) and NCI/ADR-RES-EGFP cells (**b**). Growth dynamic of the parental cells (**c**) and subline (**d**) are calculated as the fold-change in RFU relative to day 1.

**Figure S6:** **Day 12 spheroid viability curves.** On day 12, coculture spheroids treated with varying doses of NP-Tal were characterized for viability based on fluorescence of the cell lines OVCAR8-DsRed2 and NCI/ADR-RES-EGFP (**a**) and luminescence of both cell lines in the CellTiter-Glo^®^ Cell Viability Assay (**b**).


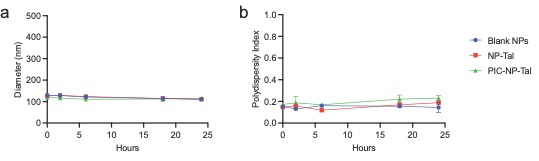


**Figure S7: Stability of nanoparticles in serum.** Polymeric nanoparticles, talazoparib-loaded nanoparticles, and polymeric nanoparticles functionalized with PIC (PIC-NP-Tal) were prepared and mixed into calcium- and magnesium-free PBS solution containing 1% fetal bovine serum (FBS). Particles were stored at 37°C and protected from light. Formulations were tracked for 24 hours and particle size (**a**) and PdI (**b**) were recorded.

**
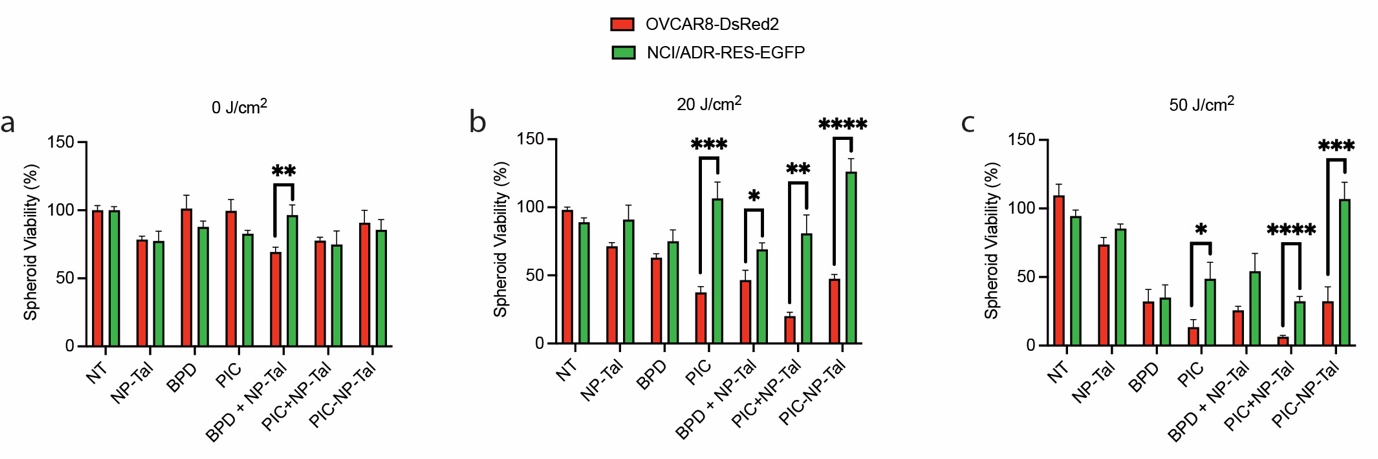
Figure S8:** **Treatment of 3D cocultures with PIC-NP-Tal.** Fluorescence-based viability of each cell line, normalized to untreated (no treatment and 0 J/cm^2^) spheroids, is shown at 0 (**a**), 20 (**b**) and 50 J/cm^2^ (**c**).
